# Osj10gBTF3-Mediated Import of Chloroplast Protein Is Essential for Pollen Development in Rice

**DOI:** 10.3389/fpls.2021.713544

**Published:** 2021-08-06

**Authors:** Xue-jiao Liu, Jiaqi Sun, Yuqing Huang, Chao Li, Peng Zheng, Yue Yuan, Hao Chen, Mehmood Jan, Huanquan Zheng, Hao Du, Jumin Tu

**Affiliations:** ^1^Institute of Crop Science, Zhejiang University, Hangzhou, China; ^2^Department of Biology, McGill University, Montreal, QC, Canada; ^3^Hangzhou Global Scientific and Technological Innovation Center, Zhejiang University, Hangzhou, China

**Keywords:** NAC protein, chloroplast import, pollen development, OsHSP82, rice

## Abstract

Chloroplasts are crucial organelles for the generation of fatty acids and starch required for plant development. Nascent polypeptide-associated complex (NAC) proteins have been implicated in development as transcription factors. However, their chaperone roles in chloroplasts and their relationship with pollen development in plants remain to be elucidated. Here, we demonstrated that Osj10gBTF3, a NAC protein, regulates pollen and chloroplast development in rice by coordinating with a Hsp90 family chaperone OsHSP82 to mediate chloroplast import. Knockout of Osj10gBTF3 affects pollen and chloroplast development and significantly reduces the accumulation of fertility-related chloroplast protein OsPPR676. Both Osj10gBTF3 and OsHSP82 interact with OsPPR676. Interestingly, the interaction between OsHSP82 and OsPPR676 is only found in the cytoplasm, while the interaction between Osj10gBTF3 and OsPPR676 also occurs inside the chloroplast. The chloroplast stroma chaperone OsCpn60 can also be co-precipitated with Osj10gBTF3, but not with OsHSP82. Further investigation indicates that Osj10gBTF3 enters the chloroplast stroma possibly through the inner chloroplast membrane channel protein Tic110 and then recruits OsCpn60 for the folding or assembly of OsPPR676. Our results reveal a chaperone role of Osj10gBTF3 in chloroplast import different from Hsp90 and provide a link between chloroplast transport and pollen development in rice.

## Introduction

Chloroplast is the center for energy production. A significant proportion of nuclear genes have been identified to be involved in the complex process of chloroplast biogenesis, such as protein translocation (Bauer et al., 2000; Motohashi et al., 2001) and assembly (Sundberg et al., 1997). Heat shock protein 90 (Hsp90), one of the largest gene families in plants, plays important chaperone activity in the import of chloroplast proteins. It has been demonstrated that cytosolic Hsp90 delivers precursor proteins to the outer chloroplast membrane receptor Toc64 by forming guidance complex with precursor proteins (Qbadou et al., 2006; Inaba and Schnell, 2008; Li and Chiu, 2010; Fellerer et al., 2011).

Nascent polypeptide-associated complex (NAC) has been reported to interact with nascent polypeptides emerged from the ribosome to prevent the inappropriate interaction of the nascent peptide with signal recognition particle in the cytoplasm (Rospert et al., 2002). NAC exists as a homodimeric NAC with two α-subunits in Archaea, and a heterodimer of α-NAC and β-NAC in other species (Preissler and Deuerling, 2012). In mammals and yeasts, NAC plays diverse roles in different biological processes, such as the developmental regulation (Deng and Behringer, 1995; Markesich et al., 2000), protein stability (Wiedmann et al., 1994; Duttler et al., 2013; Wang et al., 2013), transcription activators (Rospert et al., 2002) and protein translocation in *C. elegans* (Hotokezaka et al., 2009; Gamerdinger et al., 2015), human cells (Gamerdinger et al., 2015), and yeast (George et al., 1998; Funfschilling and Rospert, 1999; Lesnik et al., 2014; Williams et al., 2014). Furthermore, NAC has been identified as a component of ribosome-associated chaperones which promote the folding of newly synthesized proteins (Preissler and Deuerling, 2012) and the loss of NAC and HSP70 homologs result in substantial growth defects, suggesting that NAC may be connected with the chaperone system (Koplin et al., 2010).

Basic transcription factor 3 (BTF3), the β-subunit of NAC (βNAC; Wiedmann et al., 1994), was originally identified in HeLa cells as a basal transcription factor (Zheng et al., 1987) which involved in initiation of transcription from the certain class II promoters (Zheng et al., 1990). The two BTF3 homologs in *Saccharomyces cerevisiae*, EGD1 and BTT1, have been discovered to stabilize a GAL4-DNA complex (Parthun et al., 1992) and negatively regulate the expression of some galactose-regulated genes and constitutive genes, such as actin (Hu and Ronne, 1994), respectively. In addition, α-NAC has also been identified as a transcriptional coactivator for c-Jun in the nucleus (Moreau et al., 1998). These indicated the individual functions for a monomeric subunit of NAC complex. Although BTF3 (βNAC) has multiple roles in mammals, *C. elegans*, and yeasts, there is very limited knowledge about the roles of BTF3 in plants. The virus-induced gene silencing of *Nicotiana benthamiana* BTF3 (NbBTF3) reduces the chloroplast size and chlorophyll content (Yang et al., 2007), suggesting a role in chloroplast development. *Capsicum annuum* basic transcription factor 3 (CaBtf3) has been verified to regulate transcription of pathogenesis-related genes during hypersensitive response to Tobacco mosaic virus, possibly by functioning as a transcription factor (Huh et al., 2012). Recently, *Arabidopsis thaliana* BTF3 and BTF3L (BTF3-like) proteins are found to be involved in the response to cold stress through the expression regulatory of CBF genes (Ding et al., 2018). We previously shown that the inhibition of the expression of a basal transcription factor 3-like gene *Osj10gBTF3* results in plant growth defective and typical pollen abortion in rice (Wang et al., 2012). Osj10gBTF3 regulates the expression of *OsHSP82* and *OsPPR676*. OsHSP82 is a member of heat shock protein 90 (Hsp90) chaperone family. OsPPR676 is a nuclear-encoded and plastid-localized protein (Liu et al., 2017). Loss of OsPPR676 inhibits the translation of the atpB gene and reduces the activity of ATP synthase in chloroplasts, which also leads to defects in plant growth and pollen development by impairing the biosynthesis of fatty acids and starch.

In this study, we reveal a novel role of Osj10gBTF3 in the import of chloroplast proteins by using OsPPR676 as a chloroplast protein marker, contributing to a better understanding of the import mechanism of chloroplast proteins, and also provide a new insight to pollen development in rice. Furthermore, our work also revealed that plant NAC proteins can act not only as transcription factors, but also as protein chaperones.

## Materials and Methods

### Vector Construction and Rice Transformation

The allelic *hsp82-1* mutants and *btf3-1* mutants were created by the CRISPR/Cas9 mutation system in the *Nipponbare* background (*Oryza sativa L. ssp. japonica*). For transgenic rice plants expressing the fusion construct OsHSP82-RFP, the OsHSP82 CDS was cloned into a binary vector (pCAMBIA1300) under the control of the CaMV 35S promoter by homologous recombination technology. The plasmid was transformed into EHA105, and rice transformation was performed as previously described (Hiei et al., 1994). Mutant and transgenic plant identification was performed by using specific primers (Supplementary Table S1). All plants were grown in paddy fields at Hainan during the winter (28–32°C at daytime and 18–25°C at night) and Hangzhou during the normal growing seasons (25–28°C at daytime and 18–23°C at night).

### Measurement of Photosynthetic Rate

Fifteen plants were selected for each material to measure the photosynthetic rate. The leaf was first allowed to equilibrate at the ambient CO_2_ concentration of 400 μmolmol^−1^ and high light at 1,500 μmolm^−2^s^−1^ for at least 30min, and the photosynthetic parameters were recorded at 2 min after each change in the CO_2_ concentration. The photosynthetic rate was recorded using an infrared gas analyzer portable photosynthesis system (Li-Cor 6400, Lincoln, NE, United States).

### Bimolecular Fluorescence Complementation

The N-terminal amino acids of YFP were translationally fused to the N-terminus of full-length OsPPR676, and the C-terminal amino acids of YFP were translationally fused to the C-terminus of full-length Osj10gBTF3/OsHSP82 by Gateway technology. All constructs were transformed into *Agrobacterium EHA105*. Different combinations of these constructs were mixed at a 1:1 OD_600_ ratio and injected into 3–4-week-old tobacco epidermal cells. After 36 h, fluorescent signals were observed by confocal fluorescence microscopy. The plot profile in ImageJ was used for the quantification of the fluorescence intensity profiles.

### Co-immunoprecipitation Assay

For the interaction between OsHSP82 and OsPPR676, *Arabidopsis* protoplasts were isolated from 15-day leaves. The fusion construct OsHSP82/Osj10gBTF3-FLAG and OsPPR676-His were co-transformed into protoplasts for 14–16 h, respectively. Then, the total protein from the protoplast was extracted, and OsPPR676 was detected with His antibody (D110002, Sangon Biotech). OsHSP82 and Osj10gBTF3 were detected by FLAG antibody (DK3201, Elabscience).

For the interaction between OsHSP82/Osj10gBTF3 and the chloroplast translocon complex, the total proteins and chloroplast proteins from leaves of WT and transgenic plants (OsHSP82-RFP) were extracted as described previously (Cho et al., 2006; Takamatsu et al., 2018), and the soluble proteins were mixed with Osj10gBTF3 and RFP antibodies (ab62341) at 4°C overnight. Then, the protein–antibody complexes were immunoprecipitated with 200 μl protein A agarose (high affinity; ab193255) at 4°C for 4 h, and nonspecific proteins were removed by three consecutive washes with lysis buffer (20 mm Tris–HCl, pH 8.0; 137 mm NaCl; 1% Triton X-100; 2 mm EDTA; immediately before use, add protease inhibitors) every 10 min at 4°C. The protein complexes were eluted in 200 μl elution buffer (0.2 M glycine, pH 2.0; the eluted samples were immediately neutralized with Tris, pH 8.0–8.5) and subjected to Western blot analysis and visualized by Toc64 (PHY1376S), OsCpn60 (PHY0370S), and Tic110 antibody (AS08293), respectively.

### Isolation of Rice Chloroplasts

Ten-day-old plants (7.5–12 g tissue) were homogenized in 20 ml precooled isolation buffer (0.3 M sorbitol, 5 mm MgCl_2_, 5 mm EGTA, 5 mm EDTA, 20 mm HEPES/KOH, pH 7.6, 10 mm NaHCO_3_) (Aronsson and Jarvis, 2002). The homogenate was filtered through a double layer of Miracloth (Calbiochem). The debris retained was returned to beaker with 20 ml fresh isolation buffer and the homogenization was repeated. The total homogenate was centrifuged at 200 g for 5 min, 4°C, and the supernatant was transferred into a new tube and was centrifuged at 1000 g for 10 min, 4°C (acceleration: 6). Chloroplasts were resuspended in 15 ml precooled HMS buffer (0.3 M sorbitol, 3 mm MgCl_2_, 50 mm HEPES–KOH, pH 7.6) and were centrifuged at 1000 g for 5 min, 4°C. Chloroplasts were resuspended in 8 ml precooled HMS buffer and were added on 24 ml 40% Percoll solution (40% (v/v) Percoll (Solarbio P8370) in 0.3 M sorbitol, 50 mm HEPES–KOH, pH 7.6) and centrifugation at 3300 g for 20 min. Final chloroplasts were washed twice in HMS buffer and collected to extract protein.

### Subcellular Localization

The OsHSP82 cDNA was fused in frame with GFP/mCherry and inserted between the CaMV 35S promoter and the nopaline synthase (NOS) terminator in vector. The expression construct OsHSP82-GFP was transfected into rice protoplasts according to the protocols described previously (Chen et al., 2006). OsHSP82-mCherry was transformed into EHA105 and was then infiltrated into leaf epidermis of *N. benthamiana* at 600 nm (OD_600_) of 0.5–0.8 (Conley et al., 2009). The samples were observed with a confocal laser scanning microscope (Leica TCS SP5).

For subcellular localization of OsPPR676 in *hsp82-1* and *btf3-1* mutants, the OsPPR676 cDNA was fused in frame with GFP and inserted between the cauliflower mosaic virus 35S promoter and the NOS terminator in the pGWB5 vector. The expression construct was transfected into rice mutant *hsp82-1* and *btf3-1* protoplasts according to the protocols described previously (Chen et al., 2006). 35S:HDEL-RFP and 35S:RPL1-CFP were used as an ER marker and nuclear marker, respectively. The samples were observed with a confocal laser scanning microscope (Leica TCS SP5).

### Measurement Total ATP-Hydrolytic Activity of Chloroplast ATP Synthase

To measure chloroplast ATP synthase activity, chloroplast proteins were prepared from 400 mg fresh leaf samples and incubated in a 25°C water bath for 5 min, 10 min, 15 min, 20 min, and 25 min. ATP synthase activity was measured using a chloroplast ATP synthase test kit (FHTE-1-Y) from Suzhou Comin Biotechnology.

### Western Blot Analysis for Protein Stability and atpB Protein

Fifty micrograms of OsPPR676 and 50 μg of Osj10gBTF3 or OsHSP82 plasmids were co-transfected into rice protoplasts (1 × 10^5^), which were isolated from WT, *hsp82-1*, or *btf3-1* mutant seedlings at 12 days according to Guidelines for biological experiments in rice. The transformation was performed by 40% PEG and incubated in WI solution for 5 h. Samples were collected before 10 μm CHX was added, and at 3 h after 10 μm CHX was added, FLAG antibody (DK3201, Elabscience).

Chloroplast proteins were extracted from leaf tissues of 2-week-old WT, *hsp82-1*, and *btf3-1* seedlings as described previously (Cho et al., 2006). Coomassie Blue staining was used as an internal loading control. The target protein, atpB, was detected by atpB antibody (AS05085, Agrisera). The nuclear protein histone and cytoplasmic protein GAPDH were detected by histone (D151717, Sangon Biotech) and GAPDH (D110016, Sangon Biotech) antibody, respectively.

### Microscale Thermophoresis

Microscale thermophoresis (MST) is a biophysical technique that measures the strength of the interaction between two molecules by detecting variations in fluorescence signal as a result of an infrared radiation (IR) laser-induced temperature change. MST assays were conducted using a Monolith NT.115 apparatus (Nano Temper Technologies, Germany). For protein expression, Osj10gBTF3, Cpn60, and OsPPR676 expression was measured using the Expi293 suspension culture (The Expi293 expression system is a mammalian protein production system that can produce up to six times as much protein in a week as other transient systems. Due to the large amounts of protein required in MST assay, so we selected this system), purified using Ni-NTA agarose, and then subjected to gel filtration chromatography to obtain purified proteins. Target proteins (Osj10gBTF3 and OsPPR676) were labeled according to the protocol for NanoTemper His-Tag Labeling Kit RED-tris-NTA 2nd generation, PBS buffer (with 0.05% [v/v] Tween 20). For binding tests, 100 nM target protein (fluorescently labeled) was incubated with 100 nM unlabeled protein (ligand) for 10 min prior to the measurement. To determine *K*_d_, 100 nM target protein was incubated with serial dilutions of the ligand. Samples of ∼10 μl were loaded into capillaries and inserted into the MST instrument loading tray (Monolith NT.115). The thermophoresis experiments were performed using 35% MST power and 60% LED power at 24°C.

## Results

### Loss of Osj10gBTF3 Affects Pollen and Chloroplast Development in Rice

**Figure 1:**
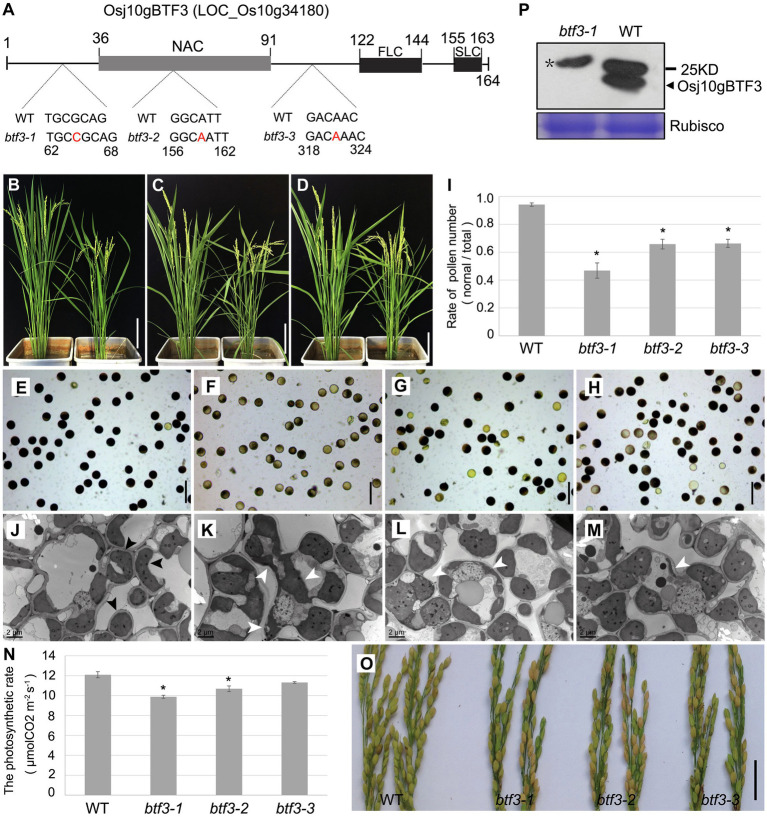
Loss of Osj10gBTF3 affects pollen and chloroplast development in rice. **(A)** Schematic of domain structure of Osj10gBTF3 and mutation sites of *btf3* mutants. FLC and SLC indicate the first and second complexity domains, respectively. **(B–D)** Comparison of plant height between WT plants (left) and the *btf3-1* **(B)**, *btf3-2* **(C)**, and *btf3-3* **(D)** mutants (right). **(E–H)** KI-I_2_ staining of pollen grains of WT **(E)** and *btf3-1* **(F)**, *btf3-2* **(G)**, and *btf3-3* **(H)** mutants. **(I)** The proportion of normal pollens checked by KI-I2-staining in WT and *btf3-1*, *btf3-2*, and *btf3-3* mutants. ^*^indicates *p* < 0.05. **(J)** to **(M)** Comparison of chloroplast in the leaves between WT and *btf3-1*, *btf3-2*, and *btf3-3* mutants (TEM). Black and white arrows indicate the normal and abnormal chloroplasts, respectively. **(N)** Comparison of photosynthetic rate between WT and *btf3-1*, *btf3-2*, and *btf3-3* mutants. Fifteen plants were selected for each material to measure the photosynthetic rate. ^*^indicates *p* < 0.05. **(O)** Comparison of panicles between WT (left) and *btf3-1*, *btf3-2*, and *btf3-3* mutants (right) at the maturing stage. Compared with WT, the number of grains with normal seeds-setting (yellow) were much fewer in *btf3-1*, *btf3-2*, and *btf3-3* mutants. **(P)** Western blot analysis of Osj10gBTF3 protein level in WT and the *btf3-1* mutant. Star(^*^) indicates the non-specific band. Scale bars: 20 cm in **(B–D)**, 100 μm in **(E–H)**, and 5 cm in **(O)**.

To further investigate the mechanism under why Osj10gBTF3 regulates the pollen development, we created three *btf3* mutant lines by the CRISPR/cas9 system (Figure 1A). Phenotype showed that the *btf3-1, btf3-2*, and *btf3-3* mutant plants were slightly shorter when compared with wild-type (WT, left; Figures 1B–D). Similar to the pollen abortion of *Osj10gBTF3^RNAi^* (Wang et al., 2012), all the three *btf3* mutant lines had different degrees of pollen abortion. More than half of the pollen of the *btf3-1* mutant were sterile, which was higher than that of the *btf3-2* and *btf3-3* mutants (Figures 1E–H). These results were also supported by the quantification of pollens (Figure 1I). To understand the subcellular defects in *btf3* mutants, we observed chloroplasts in leaves by transmission electron microscopy. Compared to the ellipsoid or spherical chloroplasts in WT (Figure 1J; black arrows), most chloroplasts in btf3-1 mutant were irregular in shape and enlarged in size (Figure 1K; white arrows), while only a small number of chloroplasts were abnormal in *btf3-2* and *btf3-3* mutants (Figures 1L,M). We also tested the rate of photosynthesis of WT and *btf3* mutants. Compared to WT, the photosynthetic rate of the *btf3-1*, *btf3-2*, and *btf3-3* mutants were all decreased with different degrees (Figure 1N). At maturing, the seed setting rate of the *btf3* mutants also decreased significantly (Figure 1O), similar to that of *Osj10gBTF3^RNAi^* (Wang et al., 2012).

Due to the more obvious pollen defect of the *btf3-1* mutant, we used this mutant for the further studies. We also examined the expression of Osj10gBTF3 using Osj10gBTF3 antibody. The Osj10gBTF3 protein was almost undetectable in *btf3-1* mutants (Figure 1P). In addition, we also knocked out the Osj10gBTF3 interacting molecular chaperone OsHSP82 (Wang et al., 2012) and found that *hsp82-1* mutant has the similar defects as *btf3* mutants (Supplementary Figure 1). These results suggest that Osj10gBTF3 may work together with OsHSP82 in the regulating chloroplast development.

### Knockout of Osj10gBTF3/OsHSP82 Reduces the Accumulation of OsPPR676 in the Chloroplast

**Figure 2:**
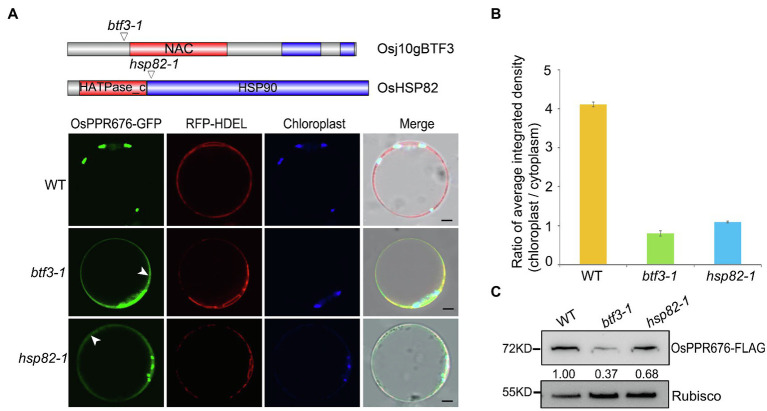
Knockout of Osj10gBTF3/OsHSP82 reduces the accumulation of OsPPR676 in the chloroplast. **(A)** Structure of the Osj10gBTF3 and OsHSP82 proteins, the corresponding mutation sites and the subcellular localization of OPPR676 in WT, *hsp82-1*, and *btf3-1* mutant rice protoplasts, respectively. The white arrows indicate the ER or cytoplasm retention signals. Scale bar = 5 μm. Two independent replicates were performed for each material. **(B)** Ratio of the chloroplast-integrated density to the cytoplasm-integrated density in WT, *hsp82-1*, and *btf3-1* mutants, respectively. Six cells were used to measure the average integrated density in each material. Values represent the mean ± SD, *n* = 3 independent replicates. **(C)** OsPPR676 protein in the chloroplast isolated from the protoplast of WT, *hsp82-1*, and *btf3-1* mutants was detected by FLAG antibody and measured quantitatively. Rubisco was used as an internal reference.

Since OsHSP82 is a typical chaperone protein and Osj10gBTF3 may cooperate with OsHSP82 in the import of nuclear-encoded proteins into chloroplasts, to investigate this hypothesis, we used the OsPPR676 protein as a chloroplast protein marker. In our previous study, OsPPR676 has been identified as a nuclear-encoded and plastid-localized protein that is essential for pollen development and confirmed to interact with Osj10gBTF3 (Liu et al., 2017). We firstly examined the subcellular localization of OsPPR676 in protoplasts of *btf3-1* and *hsp82-1* mutants. In protoplasts of WT, OsPPR676 was only seen in chloroplasts (Figure 2A; top). But it had the obvious cytosolic retention signals (white arrows) in *btf3-1* and *hsp82-1* mutants (Figure 2A; bottom 2 panels). The ratio of the chloroplast-integrated density to the cytoplasm-integrated density in *btf3-1* and *hsp82-1* mutants was also much lower than that of WT (Figure 2B). Furthermore, when the recombinant protein OsPPR676 was expressed in protoplasts of WT, *btf3-1*, and *hsp82-1* mutants, there is an obvious reduction of OsPPR676 accumulated in the chloroplasts of *btf3-1* and *hsp82-1* mutants compared with that in WT chloroplasts (Figure 2C).

Previous data showed that OsPPR676 is essential for pollen development by regulating the translation of the chloroplast atpB protein, a key subunit of ATP synthase in the chloroplast. When OsPPR676 was knocked out, the production of the chloroplast atpB protein was greatly reduced, together with the decreased activity of the ATP synthase (Liu et al., 2017). To further investigate whether Osj10gBTF3 is involved in the import of OsPPR676 into the chloroplast, we tested the protein level of atpB in the chloroplasts of *btf3-1* and *hsp82-1* mutants. As indicated in Supplementary Figure 2A, there was a significant reduction in the accumulation of chloroplast atpB in both mutants. When the ATP synthase activity was measured, there was also decreased total ATP-hydrolytic activity of the ATP synthase (Supplementary Figure 2B) in *btf3-1* and *hsp82-1* mutants compared to that in WT. The above results indicated that OsPPR676 was less accumulated in the chloroplasts of *btf3-1* and *hsp82-1* mutants, which may be resulted from the compromised import of OsPPR676 into chloroplasts.

### The Interaction Between Osj10gBTF3/OsHSP82 and OsPPR676 Is Different

**Figure 3:**
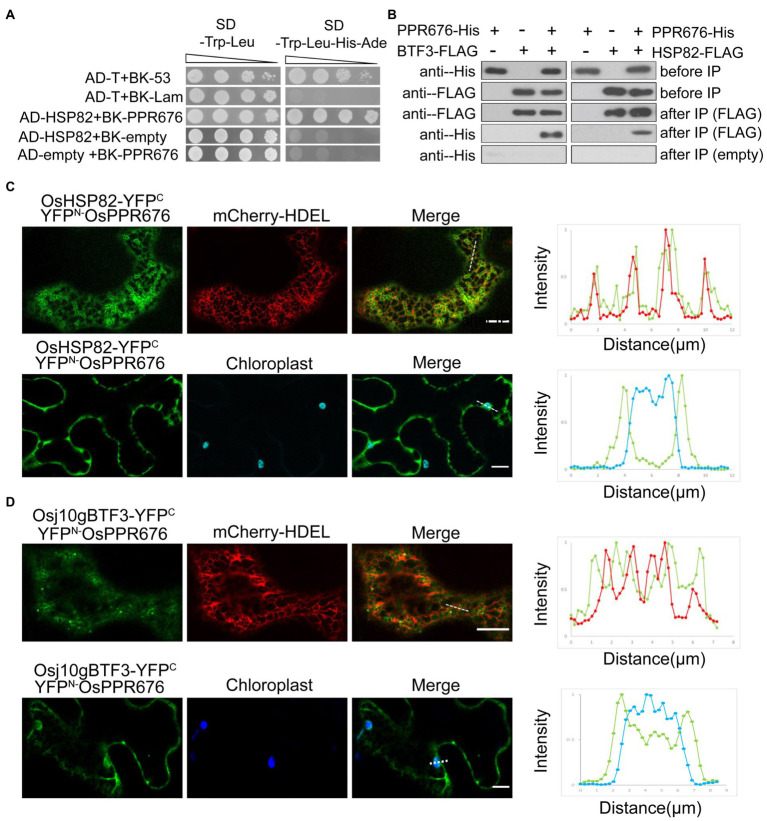
The interaction between Osj10gBTF3/OSHSP82 and OsPPR676 is different. **(A)** Y2H assays were performed to test the interaction between OsHSP82 and OsPPR676. The pGADT7-T cells co-transformed with pGBKT7-53 or the pGBKT7-Lam were used as positive or negative controls, respectively. Combinations AD-OsHSP82 with BK empty and combinations BK-OsPPR676 with AD empty were also used as negative controls. SD, Synthetic dextrose. **(B)** Co-IP assays were performed to test the interaction between Osj10gBTF3/OsHSP82 and OsPPR676 using *Arabidopsis* protoplasts. FLAG antibody conjugated agarose gel was used to co-precipitate OsPPR676. OsHSP82/Osj10gBTF3 and OsPPR676 were detected by FLAG and His antibody, respectively. Three independent replicates were performed for the experiment. **(C)** and **(D)** YFP^N^-OsPPR676 was co-transfected with either OsHSP82-YFP^C^ **(C)** or Osj10gBTF3-YFP^C^ **(D)** into the epidermal cells of tobacco. Chloroplast and mCherry-HDEL (endoplasmic reticulum marker) were used as markers. The intensity profile of the dashed line across the ER (top) or the chloroplast (bottom) in the cells was plotted for the quantification of the fluorescence intensity profiles (right). Scale bar = 10 μm. Negative controls were showed in Supplementary Figure 3.

In order to understand how Osj10gBTF3 cooperates with OsHSP82 in the import of OsPPR676 into chloroplasts, we firstly tested the interaction between Osj10gBTF3/OsHSP82 and OsPPR676. We observed the interaction between OsHSP82 and OsPPR676 by yeast-two-hybridization (Figure 3A), and OsPPR676 could also be co-purified with Osj10gBTF3/OsHSP82 (Figure 3B).

Then, we examined and quantified the distribution of the BiFC interaction signal between OsPPR676 and OsHSP82 as well as Osj10gBTF3 in *N. benthamiana* leaves. Compared with the negative controls (Supplementary Figure 3), the interaction signals were mainly in the cytoplasm and partial co-localization with ER (Figure 3C; top), but did not appear inside the chloroplast (Figure 3C; bottom). The quantification of fluorescence intensity profiles supported this notion (Figure 3C; right). For Osj10gBTF3 and OsPPR676, the interaction signals were observed mainly in the cytoplasm (Figure 3D; top). Intriguingly, we also detected some interaction signals of Osj10gBTF3 and OsPPR676 on and inside the chloroplast (Figure 3D; bottom). The quantification of fluorescence intensity profiles showed that the interaction signal on the edge of the chloroplast was stronger than the internal signal (Figure 3D; bottom, right). In addition, Osj10gBTF3 and OsPPR676 also interacted at some punctates on the ER tubules (Figure 3D). These results suggest that despite the similar cytosolic localization of interaction signals, the interacting localization of Osj10gBTF3 and OsPPR676 is different from that of OsHSP82 and OsPPR676, implying that Osj10gBTF3 may act differently from OsHSP82.

### Osj10gBTF3 and OsHSP82 Protect OsPPR676 Protein From Degradation

Considering the important roles of Osj10gBTF3 and OsHSP82 in the import of OsPPR676, we speculated that Osj10gBTF3 and OsHSP82 may protect OsPPR676 from degradation in the cytoplasm during the import of OsPPR676 into the chloroplast.

**Figure 4:**
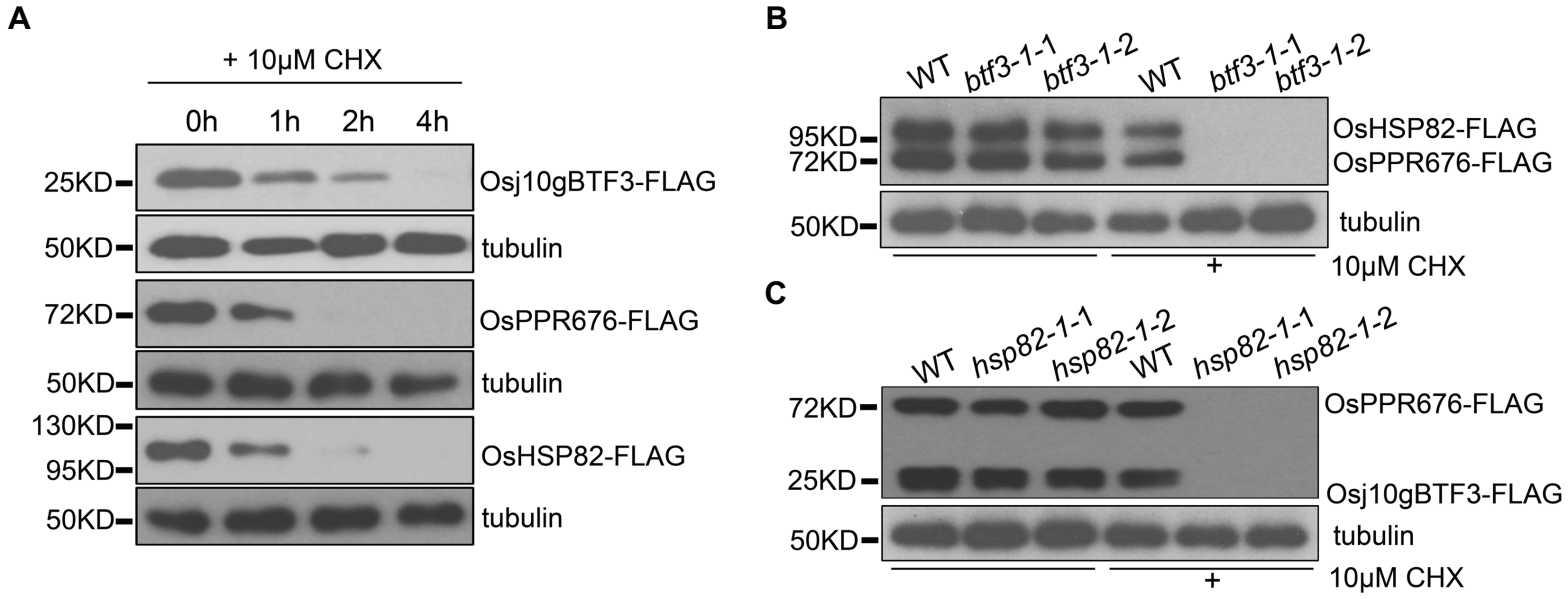
Osj10gBTF3 and OsHSP82 protect OsPPR676 protein from degradation. **(A)** Osj10gBTF3, OsHSP82, and OsPPR676 extracted from protoplasts of rice seedlings were treated with cycloheximide (CHX), respectively, and the proteins were detected by FLAG antibody at different time points. Tubulin was used as an internal reference. Three independent replicates were performed for the experiment. **(B)** OsHSP82 and OsPPR676 were co-transfected into WT and *btf3-1* mutant rice protoplasts isolated from rice seedlings, respectively. After 3 h with or without the incubation of CHX, total protein were extracted and used for Western blot. FLAG antibody was used to detect the expression of OsHSP82 and OsPPR676. *btf3-1*-1 and *btf3-1*-2 represent two duplicates. **(C)** Osj10gBTF3 and OsPPR676 were co-transfected into WT and *hsp82-1* mutant rice protoplasts isolated from rice seedlings, respectively. After 3 h with or without the incubation of CHX, total protein were extracted and used for Western blot. FLAG antibody was used to detect the expression of OsHSP82 and OsPPR676. *hsp82-1*-1 and *hsp82-1*-2 represent two duplicates.

To test this hypothesis, we carried out a series of protein stability experiments. First, the individual Osj10gBTF3, OsHSP82, or OsPPR676 vector was transfected into rice protoplasts. The protein samples were then collected at 0 h, 1 h, 2 h, and 4 h after the addition of 10 μm cycloheximide (CHX, a eukaryotic protein synthesis inhibitor) and detected by the FLAG antibody. The results showed that the three proteins were rapidly degraded after 1 h treatment with 10 μm CHX. OsPPR676 and OsHSP82 almost disappeared completely after 2 h, and Osj10gBTF3 degraded completely after 4 h (Figure 4A). The results indicated that these three proteins, when expressed individually, were all relatively unstable. We then co-transfected the OsHSP82 and OsPPR676 vectors into WT and *btf3-1* mutant rice protoplasts, respectively. Samples were collected before treatment with 10 μm CHX and after treatment for 3 h. We observed that both OsHSP82 and OsPPR676 could be detected before treatment and after CHX treatment in WT, while in the *btf3-1* mutants, both OsHSP82 and OsPPR676 could not be detected after CHX treatment (Figure 4B). These results indicated that Osj10gBTF3 plays a role in the stability of OsPPR676 and OsHSP82. Similar results were also obtained through the co-expression of Osj10gBTF3 and OsPPR676 in WT and *hsp82-1* mutant protoplasts (Figure 4C), which indicated that OsHSP82 could also protect OsPPR676 and Osj10gBTF3 from degradation. Taken all together, we conclude that Osj10gBTF3 and OsHSP82 can protect OsPPR676 from degradation in the import from the cytoplasm into the chloroplast.

### Unlike the Cytosolic OsHSP82, Osj10gBTF3 Is Localized Inside the Chloroplast As Well As the Cytoplasm

**Figure 5:**
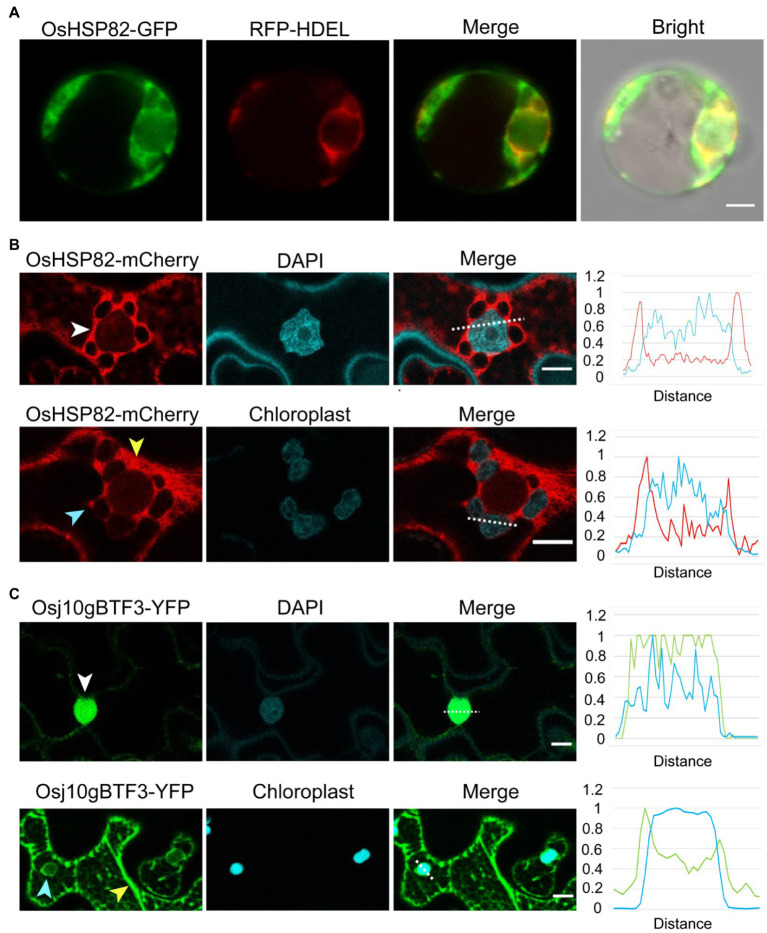
Unlike the cytosolic OsHSP82, Osj10gBTF3 is localized inside the chloroplast as well as the cytoplasm. **(A)** OsHSP82-GFP was transformed with 35S: RFP-HDEL (an endoplasmic reticulum marker) into rice protoplasts from etiolated shoots. Scale bar = 5 μm. **(B)** OsHSP82-mCherry was transformed into *N. benthamiana* leaves. The white, yellow, and light blue arrows represent the nucleus, cytoplasm, and chloroplast, respectively. The relative signal intensity of the dashed lines was quantified and presented in the right graph. Scale bar = 10 μm. **(C)** Osj10gBTF3-YFP was transformed into *Nicotiana benthamiana* leaves. The white, yellow, and light blue arrows represent the nucleus, cytoplasm, and chloroplast, respectively. The relative signal intensity of the dashed lines was quantified and presented in the right graph. Scale bar = 10 μm.

To better understand the difference between OsHSP82 and Osj10gBTF3, the subcellular localization of the OsHSP82 protein was detected by co-transformation of OsHSP82 and RFP-HDEL (an ER marker; Nelson et al., 2007) into rice protoplasts (Nelson et al., 2007; De Caroli et al., 2011). OsHSP82 was found mainly in the cytoplasm (Figure 5A).

Since the protoplasts extracted from rice etiolated seedlings are small, we also constructed a fusion protein of OsHSP82 and mCherry protein (Ransom et al., 2015) and transformed it into the leaves of *N. benthamiana* plants. Confocal microscope visualization showed that the OsHSP82 red fluorescent signal was distributed around the cell nucleus (Figure 5B; white arrow) and the chloroplast (Figure 5B; light blue arrow) as well as in the cytoplasm (Figure 5B; yellow arrow), which were also supported by the quantification of fluorescence (Figure 5B; right). We also confirmed the reported nuclear (Figure 5C; white arrow) and cytoplasmic (Figure 5C; yellow arrow) localization of Osj10gBTF3 (Wang et al., 2012). In addition, we also observed a chloroplast localization of Osj10gBTF3 (Figure 5C; light blue arrow). This chloroplast localization was further verified by the detection of Osj10gBTF3 in the chloroplast isolated from rice seedlings (Supplementary Figure 4A). We also observed that OsHSP82 and Osj10gBTF3 had only partial co-localization with the endoplasmic reticulum and main with the cytoplasm in tobacco (Supplementary Figure 4B). The different localization of Osj10gBTF3 and OsHSP82 suggests that they may have differentiated functions in terms of chloroplast import.

### Osj10gBTF3 and OsHSP82 Involve in the Import of OsPPR676 Into Chloroplasts Through Different Modes

**Figure 6:**
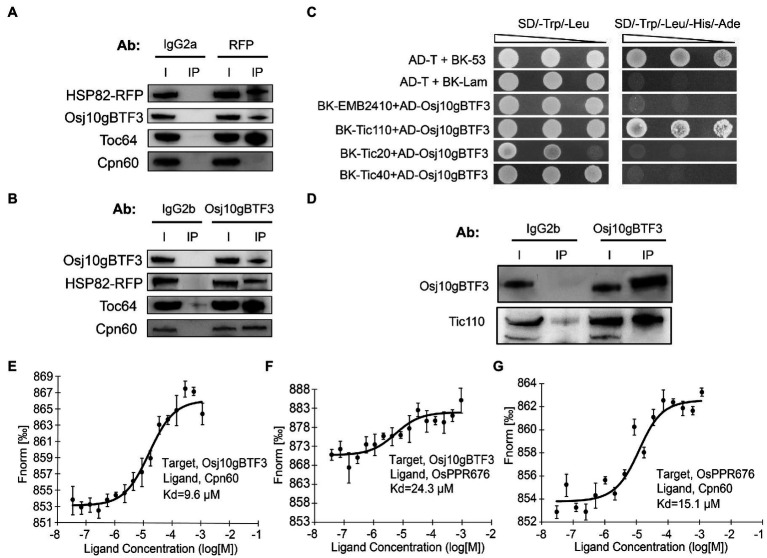
Osj10gBTF3 enters the chloroplast stroma and recruits OsCpn60 to OsPPR676. **(A)** and **(B)** Co-IP analysis of OsHSP82 **(A)** or Osj10gBTF3 **(B)** and Toc64 or Cpn60 by corresponding antibodies. Co-precipitated proteins were identified by Western blotting. The IgG2a and IgG2b were used as isotype control for RFP and Osj10gBTF3 antibodies, respectively. Ab, antibody; I, input; and IP, immunoprecipitation. Three independent replicates were performed for each experiment. **(C)** Analysis of the interaction between Osj10gBTF3 and the presumed channel proteins at the inner membrane of chloroplast. The pGADT7-T cells co-transformed with pGBKT7-53 or the pGBKT7-Lam were used as positive or negative controls, respectively. Three independent replicates were performed for the experiment. **(D)** Co-IP verification analysis of the interaction between Osj10gBTF3 and chloroplast inner membrane protein Tic110. The proteins were extracted from leaves of rice wild-type seedlings. The input is 1/4 of total loading lysate. IgG2b were used as isotype control for Osj10gBTF3 antibodies. I, input; IP, immunoprecipitation. Three independent replicates were performed for the experiment. **(E–G)** MST was performed to determine the equilibrium dissociation constants (*K*_d_) between Osj10gBTF3, OsCpn60, and OsPPR676. Values represent the mean ± SD, *n* = 3 independent replicates.

The newly synthesized precursor chloroplast proteins encoded by the nuclear genome need be imported into the chloroplast stroma through the outer and inner chloroplast membranes (Sjuts et al., 2017). It is well established that the import of cytosolically synthesized precursor proteins into chloroplasts is mediated by Toc and Tic machineries (translocon at the outer/inner envelope membrane of chloroplasts). Therefore, we tested whether OsHSP82 and Osj10gBTF3 target OsPPR676 to chloroplasts by association with the initial docking site Toc64 at the outer membrane of chloroplast. Then, co-immunoprecipitation of OsHSP82-RFP transgenic seedlings by the RFP and Osj10gBTF3 antibodies was performed, respectively. OsHSP82 and Osj10gBTF3 interacted with each other, and both showed a specific interaction with Toc64 (Figures 6A,B), while OsCpn60 specifically interacted with Osj10gBTF3, not OsHSP82 (Figures 6A,B; bottom). It has been reported that Cpn60 extracted from plant chloroplasts can effectively facilitate the folding or reconstitution of the unfold protein *in vitro* (Goloubinoff et al., 1989), This result demonstrates that Osj10gBTF3 enters into the chloroplast.

To further investigate how Osj10gBTF3 enters chloroplasts, we used Y2H assays to test the association of Osj10gBTF3 with some inner chloroplast channel proteins EMB2410 (Chen et al., 2018), Tic110 (Heins et al., 2002), Tic40 (Chou et al., 2006), and Tic20 (Reumann et al., 2005; Kovacs-Bogdan et al., 2011). We found that Osj10gBTF3 interacted with the channel protein Tic110 (Figure 6C), but not with the others. Their interaction was then verified with the Co-IP analysis (Figure 6D). These results imply that Osj10gBTF3 may enter the chloroplast stroma through Tic110.

### OsCpn60 Is Recruited by Osj10gBTF3 to the Precursor Protein

Curiously, while Osj10gBTF3 interacts with OsPPR676, the interaction appeared weaker inside the chloroplast (Figure 3D; bottom, quantification). To understand this, we measured the equilibrium dissociation constants (*K*_d_) among OsPPR676, Osj10gBTF3, and OsCpn60 to assess the intensity of the interactions (binding affinity) among them with a MST biomolecular interaction analyzer. In this assay, the smaller the *K*_d_, the stronger the interaction. Here, each combination was expressed in the Expi293 suspension culture. The results indicated that the binding affinity between Osj10gBTF3 and OsCpn60 was the strongest with the smallest *K*_d_ value (9.8 μm; Figure 6E). The binding affinity between OsPPR676 and OsCpn60 was the second, and the *K*_d_ value was 15.1 μm (Figure 6G), while the binding affinity between Osj10gBTF3 and OsPPR676 was the lowest with the largest *K*_d_ value (24.3 μm; Figure 6F). These results may imply that once OsPPR676 enters the chloroplast stroma, OsCpn60 and Osj10gBTF3 may compete to bind OsPPR676 protein.

## Discussion

Intracellular communication between the nucleus and the chloroplast is essential for the chloroplast biogenesis and function, which is important for energy homeostasis in plant cells (Guo et al., 2016). In this study, we show that Osj10gBTF3 plays a novel role in the targeting and translocation of the nuclear-encoded chloroplast protein OsPPR676 into the chloroplast, which eventually regulates pollen and chloroplast development in rice. Osj10gBTF3 not only acts as an atypical molecular chaperone in the cytoplasm to protect chloroplast precursor proteins from degradation and facilitate translocation to chloroplasts, but also may act as a novel translocon in the chloroplast stroma to transfer chloroplast precursor proteins to downstream molecular chaperones, unlike animal BTF3 that has the chaperone function. This indicated that BTF3 has evolved a new function in plants which is essential for pollen and chloroplast development.

Our work provides three lines of evidence to support our conclusion. First, the chloroplast localization of OsPPR676 is compromised in *btf3-1* and *hsp82-1* mutants (Figures 2A,B). Second, both Osj10gBTF3 and OsHSP82 protect OsPPR676 from degradation during the import of OsPPR676 to the chloroplast (Figures 4B,C). Third, mutations in OsPPR676, Osj10gBTF3, or OsHSP82 all result in decreased atpB production and ATP synthase activity (Supplementary Figures 2A,B; Liu et al., 2017).

**Figure 7:**
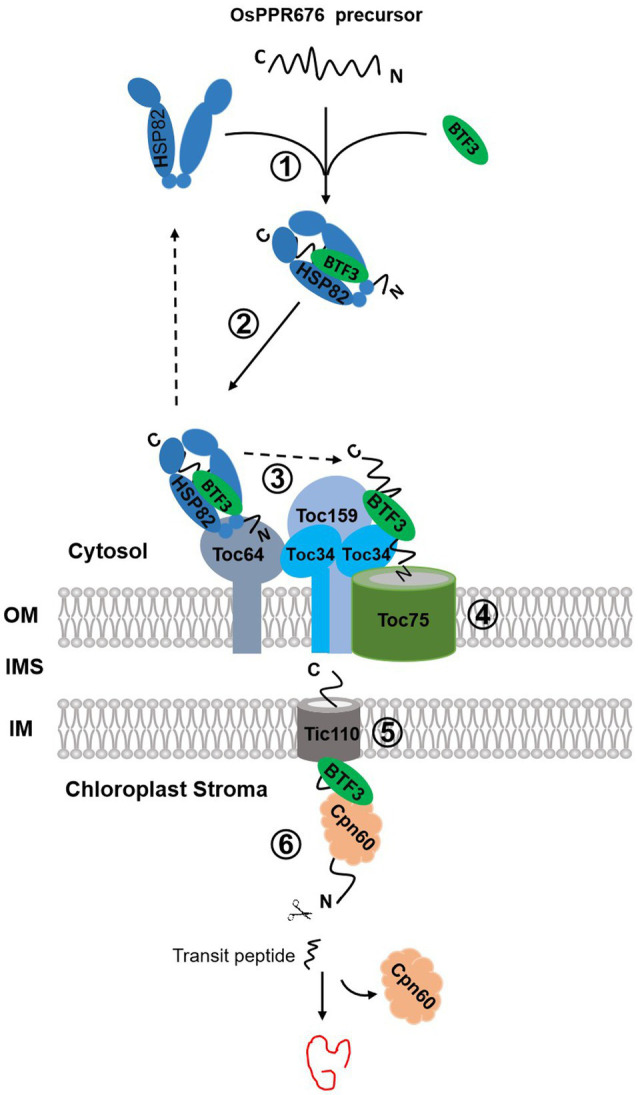
The proposed chloroplast import pathways of OsPPR676. OsPPR676, synthesized as precursor proteins in the cytosol, will form a complex with OsHSP82 and Osj10gBTF3 to prevent degradation (①). The formed OsHSP82-Osj10gBTF3-OsPPR676 complex is targeted to Toc64 *via* the interaction between Toc64 and OsHSP82 as well as Osj10gBTF3 (②). Subsequently, OsPPR676 and Osj10gBTF3 are delivered to Toc34 by a GTP-dependent association of Toc64 with Toc34 (③), with a release of OsHSP82 back to the cytosol. Next, the intrinsic GTPase activities of Toc34 and Toc159 facilitates Osj10gBTF3 and OsPPR676 to cross the outer membrane *via* Toc75 (④). After crossing the outer membrane of chloroplast, Osj10gBTF3 enters the stroma through the inner chloroplast membrane channel protein Tic110 (⑤). Once entered into the stroma, Osj10gBTF3 recruits the stromal chaperone OsCpn60 to aid the fold or assembly of OsPPR676 into a mature form or Osj10gBTF3 may be itself chaperoned by Cpn60 upon import into the chloroplast, independent of OsPPR676 (⑥). The solid arrow represents the steps supported by our data; the dotted arrow represents the steps of the data from the references. OM, outer membrane; IMS, intermembrane space; and IM, inner membrane.

We found that Osj10gBTF3, similar to OsHSP82, can physically interact with OsPPR676, and the interaction can occur mainly in the cytoplasm (Figure 3). Without this interaction, each of these proteins is degraded rapidly (Figures 4B,C). Therefore, we think that this interaction is crucial for preventing each of these proteins from degradation, possibly before OsPPR676 gets into the chloroplast, and seems to be independent of the transit peptide, because the deletion of the prediction transit peptide did not affect the interaction (data not shown). We also revealed that Osj10gBTF3 also interacts with Toc64 (Figures 6A,B). The outer chloroplast membrane receptor Toc64 has been identified as an initial docking site of molecular chaperone Hsp90 binding to chloroplast precursor proteins (Qbadou et al., 2006), which delivers the precursors into the outer chloroplast membrane channel Toc75 (Inaba and Schnell, 2008; Li and Chiu, 2010; Richardson et al., 2018). Considering that Toc64 is a specific translocon component in Hsp90-mediated chloroplast transport pathway, we suppose that Osj10gBTF3 is another crucial molecular chaperone in this pathway. It is highly likely that OsPPR676, once being made in the cytosol, will be also associated with Osj10gBTF3, this association will not only prevent the degradation of OsPPR676, but also guide and chaperone OsPPR676 to the outer chloroplast membrane *via* Toc64 (Figure 7).

It is interesting to note that, while both OsHSP82 and Osj10gBTF3 are localized to the chloroplast, OsHSP82 is only found in the periphery of the chloroplast, and Osj10gBTF3 is visible inside the chloroplasts (Figure 5; Supplementary Figure 4). It is likely that OsHSP82 and Osj10gBTF3 act in the chloroplast import of OsPPR676 differentially. The outer chloroplast membrane should be the final destination of OsHSP82, while Osj10gBTF3 may also play a role inside the chloroplast. Because Osj10gBTF3 interacts with Tic110, a channel protein in the inner chloroplast membrane (Heins et al., 2002), and stroma chaperone OsCpn60, Osj10gBTF3 may also be involved in the folding or assembly of OsPPR676 in the chloroplast stroma. Our MST biomolecular interaction analysis implies a possibility that once OsPPR676 is delivered into the stroma, Osj10gBTF3 may act as a recruiter to recruit OsCpn60 and to promote the formation of an OsPPR676-OsCpn60 complex for correctly folding or assembly of OsPPR676 in the chloroplast stroma (Figure 7), while further experiments should be done to test whether OsCpn60 competes with Osj10gBTF3 on the same binding site of OsPPR676. In this study, we focused on the chaperone function of Osj10gBTF3 and OsHSP82. OsPPR676 was only used as a chloroplast protein marker to investigate the chaperone roles of Osj10gBTF3. OsPPR676 has been identified as plastid-localized protein that is essential for pollen development and OsPPR676 interacts with Osj10gBTF3 (Liu et al., 2017) and OsHSP82. The Hsp90 family (Fellerer et al., 2011) and NAC (Gamerdinger et al., 2015) have been reported to be capable of chaperoning different precursor proteins to organelles in the cytoplasm. As a molecular chaperone in import system, we think that it is likely to be functionally universal for at least the same type of protein.

Recently, Blanco et al. demonstrated that in plant cells, the kinase SnRK1.1, a catalytic subunit of the SnRK1 complex, acts as a sensor of cellular energy status by integrating energy and stress signals from chloroplasts (Blanco et al., 2019). Based on the chloroplast energy regulation of Osj10gBTF3/OsHSP82 and OsPPR676, we detected that OsSnRK1.1 can phosphorylate OsPPR676 *in vitro* and *in vivo* and regulates the interaction between Osj10gBTF3/OsHSP82 and OsPPR676 (data not shown), suggesting that OsSnRK1.1 may play a regulatory role in the chloroplast import to be involved in the energy regulation. It will be interesting to investigate the roles of OsSnRK1.1 in chloroplast biogenesis.

## Data Availability Statement

The original contributions presented in the study are included in the article/Supplementary Material, and further inquiries can be directed to the corresponding authors.

## Author Contributions

JT initiated the project. X-JL performed most of the experiments and wrote the manuscript. JS carried out the subcellular localization and BiFC assay. HD completed the Co-IP and affinity test assay and provided suggestion on revision. HZ, JS, and JT provided guidance in experimental performance, data interpretation, and manuscript finalization. YH, CL, PZ, HC, YY, and MJ assisted in experiments. All authors contributed to the article and approved the submitted version.

## Conflict of Interest

The authors declare that the research was conducted in the absence of any commercial or financial relationships that could be construed as a potential conflict of interest.

## Publisher’s Note

All claims expressed in this article are solely those of the authors and do not necessarily represent those of their affiliated organizations, or those of the publisher, the editors and the reviewers. Any product that may be evaluated in this article, or claim that may be made by its manufacturer, is not guaranteed or endorsed by the publisher.
