# Supplementary material for: Osj10gBTF3-Mediated Import of Chloroplast Protein Is Essential for Pollen Development in Rice
Source: Front Plant Sci. 2021 Aug 6;12:713544. doi: 10.3389/fpls.2021.713544 (PMC8377413; doi:10.3389/fpls.2021.713544)
Supplement: Supplementary file 1 [file Data_Sheet_1.docx]

**Osj10gBTF3-mediated import of chloroplast protein is essential for pollen development in rice**

**Supplementary Figures**


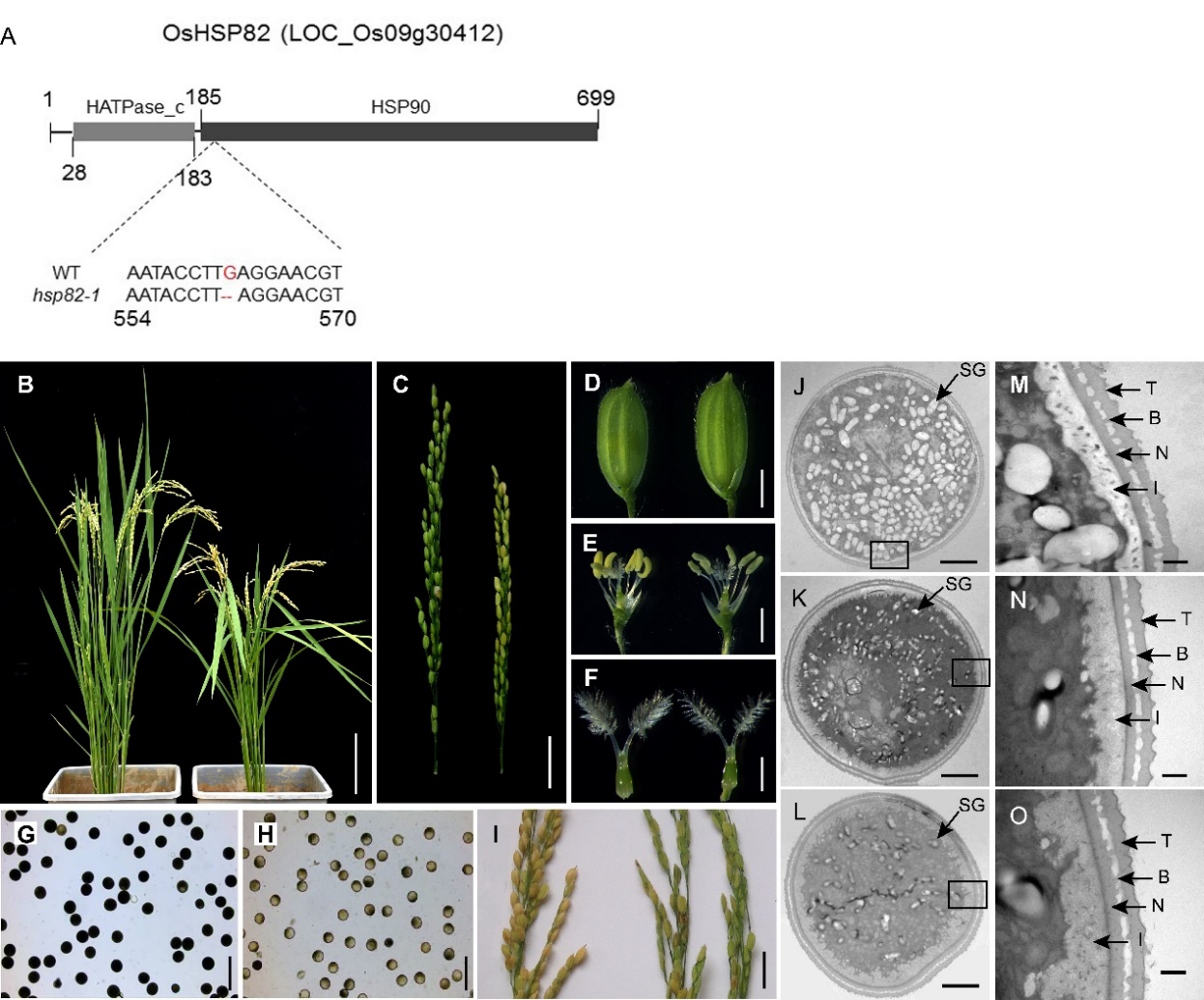


**Supplementary Figure 1.** **Phenotypic analysis of the *hsp82-1* mutant.**

**(A)** Schematic of domain structure of OsHSP82 and the mutation type of *hsp82-1.*

**(B)** WT plants (left) and the *hsp82-1* mutant (right) and after flowering.

**(C)** The panicles of the WT (left) and *hsp82-1* mutant (right) at the filling stage.

**(D)** to **(F)** The spikelets **(D)**, anthers **(E)** and pistils **(F)** of WT (left) and the *hsp82-1* mutant (right) at the flowering stage.

**(G)** and **(H)** KI-I_2_ staining of WT **(G)** and *hsp82-1* mutant **(H)** pollen, respectively.

**(I)** The panicles of WT (left) and the *hsp82-1* mutant (right) at the harvest stage.

**(J)** to **(L)** Transmission electron microscopy image of pollen grain of WT and two *hsp82-1* mutant lines.

**(M)** to **(O)** Higher magnification images of the pollen walls were shown in **(J)**, **(K)**, and **(L)**, respectively. SG, starch grain; T, tectum; B, bacula; N, nexine; I, intine. Scale bars: 20 cm in **(B)**, 3 cm in **(C)**, 2 mm in **(D)**, 1 mm in **(E)** an**d** **(F)**, 100 μm in **(G)** and **(H)**, 5 cm in **(I)**, 5 μm in **(J)** to **(L)**, 500 nm in **(M)** to **(O)**.


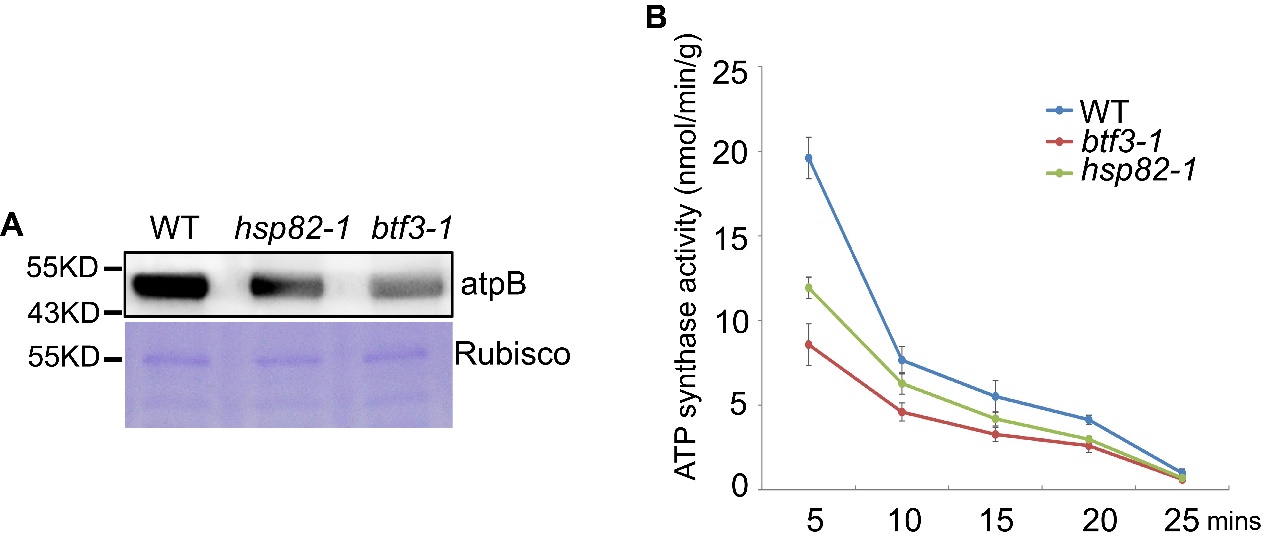


**Supplementary Figure 2.** **Effect of OsHSP82 and Osj10gBTF3 on the accumulation of atpB protein and the activity of ATP synthase.**

**(A)** Chloroplast proteins from WT and *hsp82-1* and *btf3-1* mutant seedlings were measured with atpB antibody and Coomassie Blue staining was used to indicate the sample loading amount.

**(B)** Total ATP-hydrolytic activity of ATP synthase was measured in the chloroplasts of WT and the *hsp82-1* and *btf3-1* mutants. Error bars indicate the SE based on three biological replicates.

**
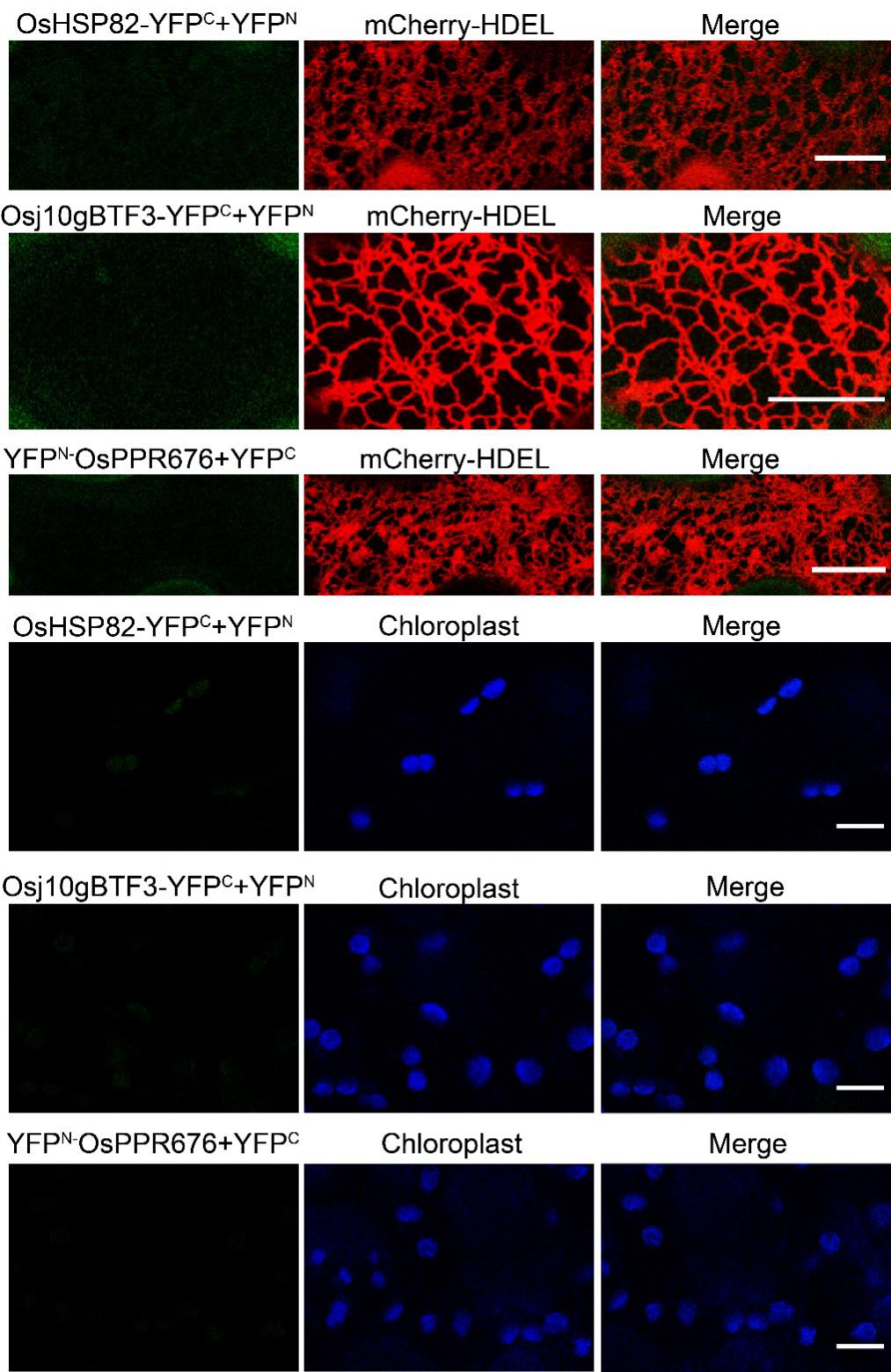
**

**Supplementary Figure 3.** **Negative controls of BiFC assay in Figure 3.**

The negative combination OsHSP82-YFP^C^ and YFP^N^ empty, Osj10gBTF3-YFP^C^ and YFP^N^ empty, and YFP^N^-OsPPR676 and YFP^C^ empty were co-transformed into leaf epidermal cells of *N. benthamiana*, respectively. The mCherry-HDEL represents the endoplasmic reticulum marker, Chloroplast indicates auto-fluorescence of chloroplasts. Scale bar=10μm.

**
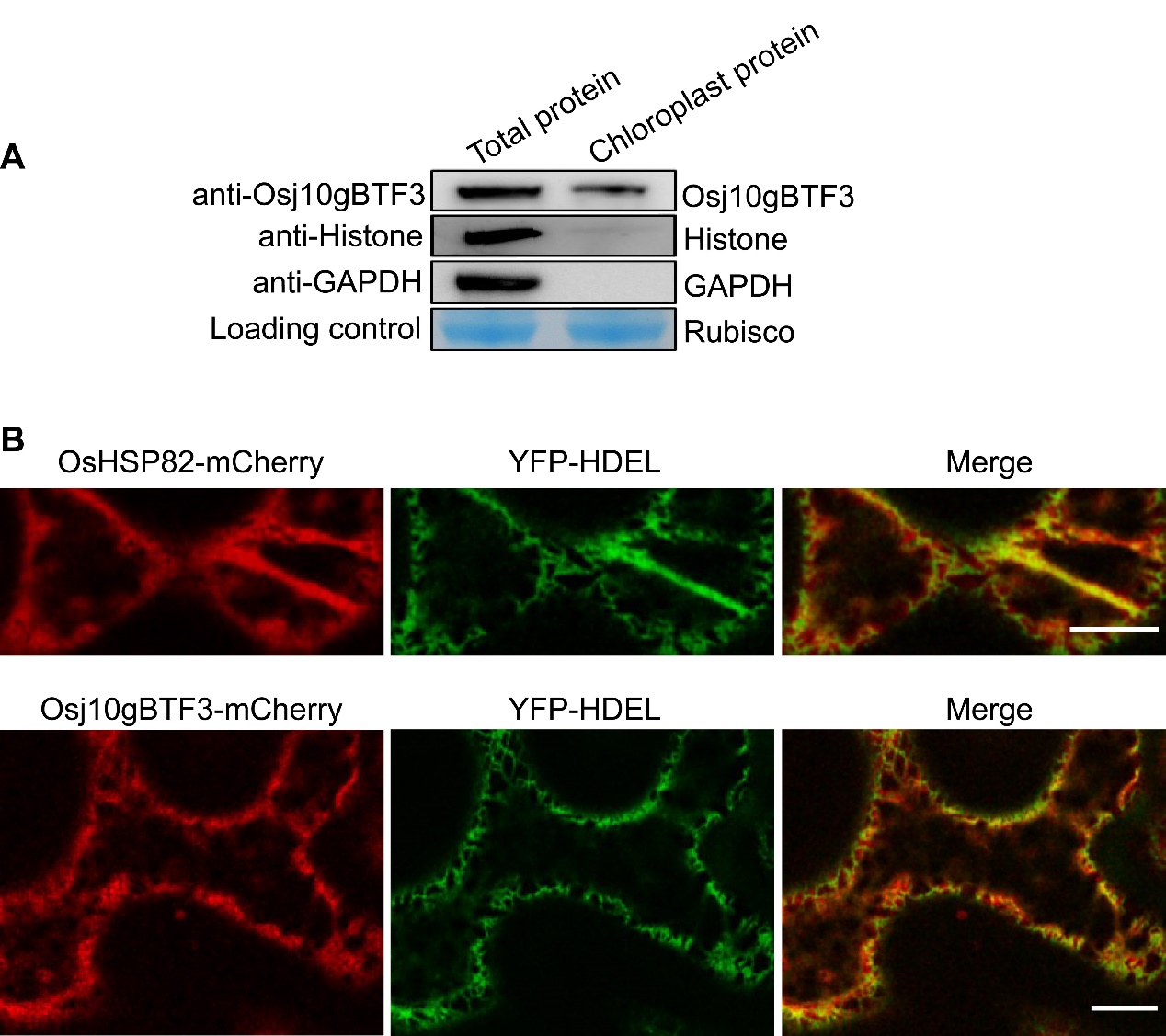
**

**Supplementary Figure 4.** **The localization of Osj10gBTF3 and OsHSP82.**

**(A)** The chloroplast localization of Osj10gBTF3.Total protein and the chloroplast protein were extracted from rice seedlings, the nuclear protein Histone and cytoplasmic protein GAPDH were used as negative control.

**(B)** The localization of Osj10gBTF3 and OsHSP82 in tobacco. YFP-HDEL is an endoplasmic reticulum marker.

**Table S1.** Main primer sequences.

| Primer name | Primer Sequence (5' to 3') | Use for |
| --- | --- | --- |
| HSP82-Y2H-F1 | GGAATTCCATATGATGGCGTCGGAGACCGA | Yeast two-hybrid |
| HSP82-Y2H-R2 | TCCCCCGGGTTAGTCGACCTCCTCCATC | Yeast two-hybrid |
| PPR676-Y2H-F1 | CCGGAATTCATGGCTTCCCCTTCCTCCCT | Yeast two-hybrid |
| PPR676-Y2H-R | ACGCGTCGACCACCGCTACTAACTCTGCCG | Yeast two-hybrid |
| HSP82-ENF | CACCATGGCGTCGGAGACCGA | BiFC |
| HSP82-ENR | GTCGACCTCCTCCATC | BiFC |
| OsPPR676-ENF1 | CACCATGGCTTCCCCTTCCTCCCT | BiFC |
| OsPPR676-ENR1 | CACCGCTACTAACTCTGCCG | BiFC |
| HSP82-F | GCTCTAGAATGGCGTCGGAGACCGA | Subcellular localization |
| HSP82-R | GCTCTAGAGTCGACCTCCTCCATCTTGC | Subcellular localization |
| GP1095-F | CCACCAAGCACAACGACGAC | Confirm *hsp82-1* mutant |
| GP1095-R | CCTTGGTGATCTCCTCTGGC | Confirm *hsp82-1* mutant |
| GP1101-F | ACACATGCATCAAAATGCACA | Confirm *btf3-1* mutant |
| GP1101-R | TTGAACCTGCTGTTGTTGGC | Confirm *btf3-1* mutant |
